# HPLC–MS/MS Analysis of a Traditional Chinese Medical Formulation of Bu-Yang-Huan-Wu-Tang and Its Pharmacokinetics after Oral Administration to Rats

**DOI:** 10.1371/journal.pone.0043848

**Published:** 2012-08-29

**Authors:** Lee-Hsin Shaw, Lie-Chwen Lin, Tung-Hu Tsai

**Affiliations:** 1 Institute of Traditional Medicine, National Yang-Ming University, Taipei, Taiwan; 2 National Research Institute of Chinese Medicine, Taipei, Taiwan; 3 Graduate Institute of Acupuncture Science, China Medical University, Taichung, Taiwan; 4 Department of Education and Research, Taipei City Hospital, Taipei, Taiwan; Queen Elizabeth Hospital, Hong Kong

## Abstract

Bu-yang-huan-wu-tang (BYHWT) is one of the most popular formulated traditional Chinese medicine prescriptions, and is widely for prevention of ischemic cardio-cerebral vascular diseases and stroke-induced disability. A specific high-performance liquid chromatography-tandem mass spectrometry (HPLC-MS/MS) has been developed and validated for simultaneous quantification of the nine main bioactive components, i.e., astragaloside I, astragaloside II, astragaloside IV, formononetin, ononin, calycosin, calycosin-7-O-β-d-glucoside, ligustilide and paeoniflorin in rat plasma after oral administration of BYHWT extract. This method was applied to investigate the pharmacokinetics in conscious and freely moving rats. No significant matrix effects were observed. The overall analytical procedure was rapid and reproducible, which makes it suitable for quantitative analysis of a large number of samples. Among them, three astragalosides and four isoflavones in A. *membranaceus*, ligustilide in Radix Angelicae Sinensis and Rhizoma Ligustici Chuanxiong and paeoniflorin in Radix Paeoniae Rubra were identified. This developed method was then successfully applied to pharmacokinetic studies of the nine bioactive constituents after oral administration of BYHWT extracts in rats. The pharmacokinetic data demonstrated that astragaloside I, astragaloside II, astragaloside IV and ligustilide presented the phenomenon of double peaks. The other herbal ingredients of formononetin, ononin, calycosin, calycosin-7-O-β-d-glucoside and paeoniflorin appeared together in a single and plateau absorption phase. These phenomenona suggest that these components may have multiple absorption sites, regulation of enterohepatic circulation or the gastric emptying rate, or there is ingredient-ingredient interaction. These pharmacokinetic results provide a constructive contribution to better understand the absorption mechanism of BYHWT and to support additional clinical evaluation.

## Introduction

BYHWT is one of the most popular formulated traditional Chinese medicine (TCM) prescriptions, with wide use in clinical medication for the treatment and prevention of ischemic cardio-cerebral vascular diseases and stroke-induced disability. BYHWT was originally recorded in the traditional herbal text *Yi-Lin-Gai-Cuo* (Chinese mean: corrections of errors among physicians) from the Qing Dynasty, published in 1830. From the viewpoint of traditional Chinese medicine, it was used to invigorate the body, promote blood circulation, and activate energy (*qi*) flow through energy meridians. Considerable clinical evidence has indicated that the ameliorative effects of BYHWT on coronary heart disease with *qi* deficiency and blood stasis syndrome in rats are associated with the inhibition of C-reactive protein and the CD40 gene, as well as the regulation of endothelium-derived vasoactive factors [1]; and mediating by the improvement of hemorheological disorders and energy metabolism [2]. A previous study has found that BYHWT could protect mice against ischemic stroke and extend lifespan, primarily through a significant down-regulation of genes involved in inflammation, apoptosis, angiogenesis and blood coagulation, as well as an up-regulation of genes mediating neurogenesis and nervous system system development [3]. Another recent study showed that the neuroprotective mechanism was associated with the down-regulation of metabotropic glutamate receptor-1 RNA and the inhibition of glutamate release resulting from cerebral ischemia [4].

BYHWT is one of the most classical medicinal prescriptions, comprised of seven commonly used Chinese herbs. According to the *Chinese Pharmacopoeia* (2005), the formula includes the following 7 herbs: (1) Radix Astragali (*huangqi*), the dried roots of *Astragalus membranaceus* (Fisch.) Bge.var. *mongholicus* (Bge.) Hsiao; (2) the carda part of Radix Angelicae Sinensis root (*guiwei*), the dried lateral roots of *Angelica sinensis* (Oliv.) Diels; (3) Radix Paeoniae Rubra (*chishao*), the dried roots of *Paeonia lactiflora* Pall.; (4) Rhizoma Chuanxiong (*chuanxiong*), the dried rhizomes of *Ligusticum chuanxiong* Hort; (5) Flos Carthami (*honghua*), the dried flowers of *Carthamus tinctorius* L.; (6) Semen Persicae (*taoren*), the dried seeds of *Amygdalus persica* L.; and (7) Pheretima (*dilong*), the dried bodies of *Pheretima aspergillum* (E. Perrier), in the ratio of 120∶6∶4.5∶3∶3∶3∶3 on a dry weight basis, respectively. In cases such as this, the combined effects of herbs in a multi-herbal formula may produce a powerful curative action.

Based on the theories of traditional Chinese medicine, a traditional herbal formulation contains more than single Chinese herb. Generally speaking, a traditional herbal formulation such as BYHWT is prescribed according to the principle of monarch, minister, assistant and guide. The monarch of Radix Astragali acts as the chief drug for treating the disease; the minister of Radix Angelicae Sinensis root serves to intensify the effect of the monarch drug; the assistant of Radix Paeoniae Rubra, Rhizoma Chuanxiong, Flos Carthami and Semen Persicae helps to deal with the secondary symptoms or inhibit the toxicity of the monarch drug; and the guide drug of Pheretima leads the other herbs to the diseased parts and balances the effects of all herbs.

According to the ratio of 120∶6∶4.5∶3∶3∶3∶3 on the dry herbal weight of BYHWT, Radix Astragali is the most abundant content of BYHWT and also a frequently used Chinese herb for oriental medicine. Radix astragali mainly contained the components such as saponins, isoflavone, polysaccharide and amino acid [5]. Pharmacological studies and chemical investigations of Radix Astragali have found that flavonoids and saponins are the two main types of beneficial compounds responsible for its pharmacological activities and therapeutic efficacy [6] [7]. Both saponins and flavonoids should be considered as marker compounds for the chemical evaluation of Radix Astragali [5]. In the present study, astragaloside IV was the marking compound of Radix astragali for quality identification, and three main astragalosides such as astragaloside I, astragaloside II and astragaloside IV were selected for analysis. Thus, the qualitative and quantitative analysis of astragalosides should be observed.

Major flavonoids in Radix astragali were formononetin, ononin, calycosin and its glycoside [8], which boost energy, strengthen the immune system, promote health activities and promote skin growth [9]. Ligustilide, a phthalide derivative, is the most abundant constituent in the herb and was also the most abundant bioactive ingredient in Radix Paeoniae Rubra and Rhizoma Chuanxiong. Vasodilatation, antiplatelet aggregation, antithrombotic, serotonergic activity, and antiproliferative properties of ligustilide have been well documented [10] [11]. Paeoniflorin was the marking compound in Radix Paeoniae Rubra. The effects were inhibited the fluorescent intensity of intracellular Ca2+ and the activities of mitogen-activated protein kinase and protein kinase C [12]. Hypoxanthine was the major marker component in Pheretima aspergillum (Di Long). However, in our preliminary experiments, hypoxanthine could not be observed in rat plasma after oral administration of BYHWT.

We expected that several major compounds of BYHWT extract would be detected in rat plasma after oral administration. Based on a pilot study, we finally detected nine BYHWT-related compounds in rat plasma. It can be observed that astragaloside I, astragaloside II, astragaloside IV, formononetin, ononin, calycosin, calycosin-7-O-β-d-glucoside, ligustilide and paeoniflorin were the main bioactive compounds in rat plasma after oral administration of BYHWT extract.

Several analytical methods have been developed for quantification of the target components in BYHWT, such as HPLC-DAD-ELSD coupled with HPLC-TOF/MS [13] [14]. However, there are few reports available regarding pharmacokinetic studies of BYHWT. One report has previously described the analysis of multiple components of BYHWT in a pharmacokinetic study, with the determination of formononetin, ononin, calycosin, calycosin-7-O-β-d-glucoside and paeoniflorin by LC-MS [15]. However, dealing with just five constituents from one or two medicinal herbs is not corresponding with the characteristics of exhibiting effects on multiple targets. The multicomponent analysis of BYHWT, however, is not easy due to the complexity of its components.

To the best of our knowledge, no LC-MS/MS method has been reported for the simultaneous estimation of multiple constituents (i.e., flavonoids and saponins) in a single run of a conscious and freely moving rat for pharmacokinetic investigation. As a common analytical tool for various compounds, LC–MS/MS provides several advantages over LC-MS in terms of sensitivity, selectivity, etc. In this study we describe an HPLC-MS/MS assay with multiple reaction monitoring (MRM) for the measurement of nine compounds in rat plasma. This analytical method using MRM was expected to be more specific and sensitive than single-ion monitoring (SIM). Therefore, in order to simultaneously characterize and identify astragaloside I, astragaloside II, astragaloside IV, formononetin, ononin, calycosin, calycosin-7-O-β-d-glucoside from Radix Astragalis; ligustilide from Radix Angelicae Sinensis and Rhizoma Ligustici Chuanxiong and paeoniflorin from Radix Paeoniae Rubra, an LC-MS/MS experiment was employed to examine the specific fragmentation patterns. The applicability of these methods in assessing plasma bioactive compound following oral administration of BYHWT extract to the pharmacokinetic study in rat is also described. The pharmacokinetic study of the above components could help to elucidate the absorption mechanism of BYHWT for additional interpretation of traditional Chinese medicine. It also could help to evaluate the effectiveness of this prescription and provide comprehensive quantification to ensure accuracy in clinical application.

## Materials and Methods

### Reagents

The authentic standards of astragaloside I, astragaloside II, formononetin, calycosin, and calycosin-7-O-β-d-glucoside were purchased from the Shanghai Tauto Biotech Co. LTD. (China) and stored at 4°C. The standard for ononin was obtained from Extrasynthese (France). Ligustilide was supplied by ChromaDex. Inc. USA (10 mg/mL in acetonitrile). Paeoniflorin was from Nacalai tesque, Inc. (Kyoto, japan). Astragaloside IV, digoxin (internal standard), pentobarbital and heparin were provided by Sigma-Aldrich Chemicals (St. Louis, MO, USA). Ammonium acetate, formic acid, acetonitrile, and methanol were of HPLC grade for analysis grade from E. Merck (Darmstadt, Germany). Triply deionized water from Millipore (Bedford, MA, USA) was prepared for all aqueous solutions.

### Herbal preparation of bu-yang-huan-wu-tang

The crushed herbs of Radix Astragali (1800 g), Radix Angelicae sinensis (90 g), Radix Paeoniae rubra (90 g), Rhizoma Chuanxiong (45 g), Semen Persicae (45 g), Flos Carthami (45 g), and Phizoma Chuanziong (45 g) were purchased from a Chinese traditional herbal medicine store in Taipei and prepared in the National Research Institute of Chinese Medicine, Taipei, Taiwan. The herbal admixture was decocted with 50% ethanol in a water bath (70°C, 18 L, 3 times) for 9 h and the least volume of solvent was added to the given aliquot. The extracted solution was evaporated under vacuum and partitioned. Water was removed by freeze drying. The yield of the crude extract of BYHWT was 366 g. The lyophilized powder of BYHWT was used for the following experiment.

**Figure 1:**
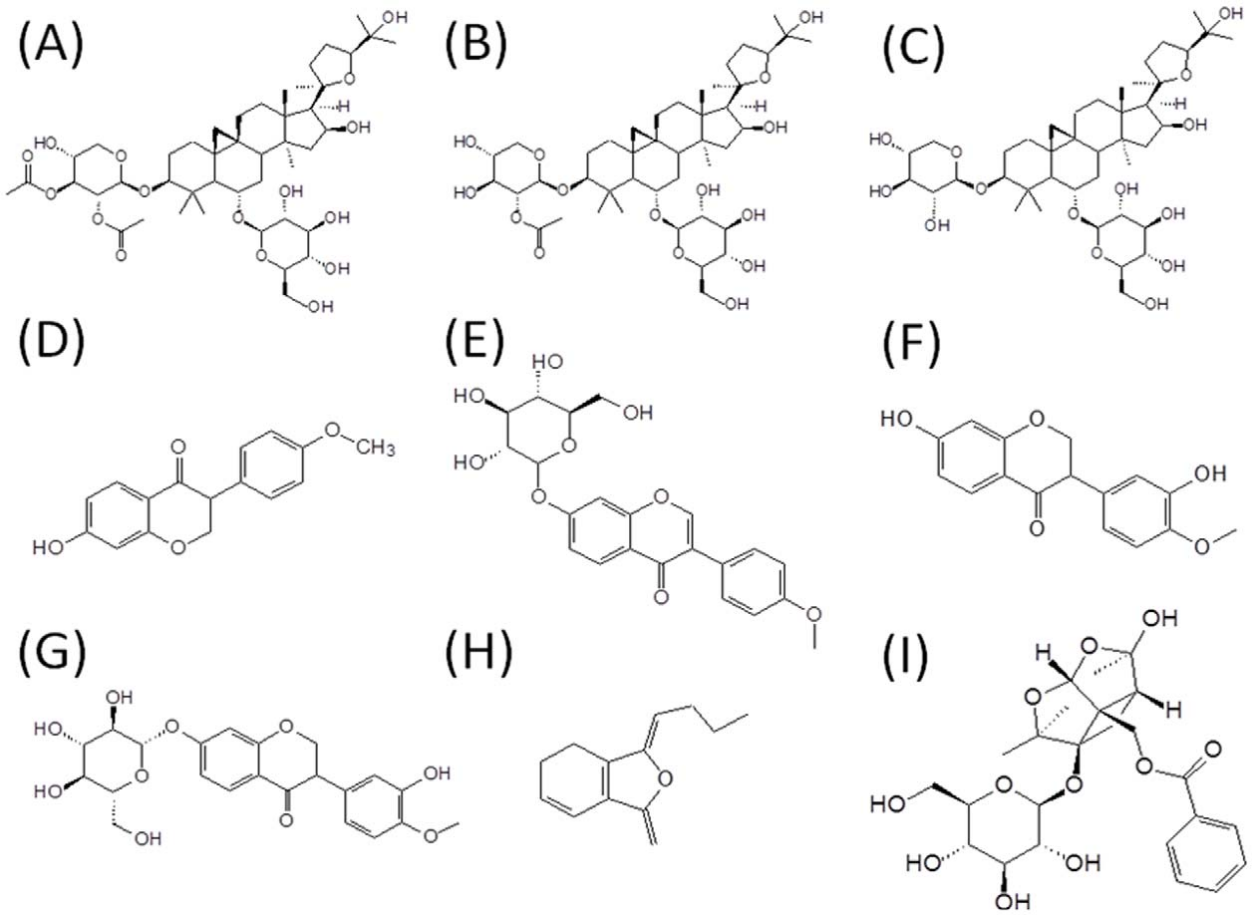
Structure formula of 9 components in bu-yang-huan-wu-tang extract of astragaloside I (A), astragaloside II (B), astragaloside IV (C), formononetin (D), ononin (E), calycosin (F), calycosin-7-O-β-D-glucoside (G), ligustilide (H) and paeoniflorin (I).

Content of astragaloside I, astragaloside II, astragaloside IV, formononetin, ononin, caycosin, calycosin-7-O-β-d-glucoside, ligustilide and paeoniflorin in BYHWT To calculate the administration dosage, the contents of astragaloside I, astragaloside II, astragaloside IV, formononetin, ononin, caycosin, calycosin-7-O-β-d-glucoside, ligustilide and paeoniflorin in BYHWT were determined by HPLC external standard method with the analytical method (section 2.5) used in this study. The results demonstrated that the content of the constituents were quantitated in BYHWT as follows (mg/g): astragaloside I 2.46 mg/g, astragaloside II 0.69 mg/g, astragaloside IV 0.54 mg/g, formononetin 2.44 mg/g, ononin 0.63 mg/g, caycosin 0.68 mg/g, calycosin-7-O-β-d-glucoside 2.56 mg/g, ligustilide 1.08 mg/g and paeoniflorin 3.11 mg/g. The structures of astragaloside I, astragaloside II, astragaloside IV, formononetin, ononin, caycosin, calycosin-7-O-β-d-glucoside, ligustilide and paeoniflorin were shown in Fig. 1.

### Freely-moving rat model

The protocol listed below has been reviewed and approved by the Institutional Animal Care and Use Committee (IACUC; approval number 1010409) by the Institutional Animal Experimentation Committee of the National Yang-Ming University. Male pathogen-free Sprague-Dawley rats (250±50 g body weight) were obtained from the Laboratory Animal Center of National Yang Ming University. The animals had free access to food (Laboratory rodent diet 5P14, PMI Feeds, Richmond, IN, USA) and water was allowed at all times. All animal experiments followed the National Yang-Ming University guidelines and procedures for the care of laboratory animals.

A freely-moving rat model was applied to minimize the stress caused by restraint or anesthesia [16]. Experimental rats were initially anesthetized with pentobarbital (50 mg/kg, i.p.), and polyethylene tubing was implanted in the right jugular vein for blood sampling. The tube crossed the subcutaneous tissue and was fixed at its exit from the dorsal neck region. The patency tubing was maintained by filling with heparinized saline (20 units/mL). After surgery, the rats were placed in experimental cages and allowed to recuperate for one day. The rats were kept fasted for 12 h and water were available ad libitum before the BYHWT administration.

### Oral administration and sample preparation

BYHWT extract was dissolved in normal saline at doses of 10 g/kg for oral administration and fed by gavages to rats, respectively. The dosage of BYHWT was selected by the indication of concentrated herbal extracts (powdered) for clinical application which was 3.5 g each time and 3–4 times daily in adults. According to the dose translation from animal to human studies [17], we used the body surface area (BSA) normalization method to convert the dose of nine active compounds for translation from humans to rat. In our study, the dose of BYHWT (10 g/kg) was appropriate for oral administration in rats. An aliquot of 100–120 µL blood sample was withdrawn from the jugular vein into a heparin-rinsed vial with a fraction collector according to a programmed schedule at 5, 15, 30, 45, 60, 90, 120, 150, 180, 240, 300, 360, 480, 600 and 720 min. The condensed blood collection in the absorption phase is to figure out the maximum concentration and the time when it reaches the maximum concentration. The time points in the elimination phase are not close. This strategy is commonly used in the pharmacokinetic studies but the total blood consumption should not be over 20% of body blood. In these experimental rats, the total blood sampling volume is within 20% during 12 h [18]. Plasma was separated by centrifuging the blood sample at 6000 rpm for 10 min at 4°C. The plasma samples (50 µL) were vortex-mixed with double volume (100 µL) of acetonitrile containing digoxin (100 ng/mL) as the internal standard for protein precipitation. The samples were vortexed and centrifuged at 16,100×g for 10 min. In our micro-analysis, less than 200 µL is needed. Actually, the collected blood volume is around 120–150 µL for each time point. After centrifugation, the plasma samples (50 µL) were vortex-mixed with 100 µL of acetonitrile. The supernatants were collected and filtered. Around 20 µL aliquot of the supernatants was transferred into the vial for analysis by HPLC-MS/MS system. The acetonitrile but not the real sample was used in the needle pre-wash standard procedure [18] [19].

### Liquid chromatography–tandem mass spectrometry

The LC–MS/MS system consisted of an Agilent 1100 series LC with automatic liquid chromatographic sampler, injector, bi-pump and degasser; and Applied Biosystems/MDS Sciex API 3000 tandem quadrupole mass spectrometry equipped with electrospray ionization (ESI) interface using the following parameters: nebulizer gas (NEB):10, curtain gas (CUR): 7, collisionally activated dissociation gas (CAD): 12, ionspray voltage (IS): 5000 V, turbo ionspray temperature (TEM): 450°C. Nitrogen was used in all cases. The mass spectrometer was operated in the positive ion detection mode. Analytes were quantified by multiple-reaction monitoring (MRM) mode performing the following precursor-to-product ion pairs of the transitions m/z: m/z 886.6 [M+NH_4_]^+^→m/z 143.2 for astragaloside I (declustering potential 55 V, focusing potential 290 V, entrance potential 12 V, collision energy 23 V, collision cell exit potential 13 V); m/z 844.5 [M+NH_4_]^+^→m/z 143.2 for astragaloside II (DP 62 V, FP 330 V, EP 13 V, CE 25 V, CXP 13 V); m/z 802.6 [M+NH_4_]^+^→m/z 143.2 for astragaloside IV (DP 65 V, FP 370 V, EP 14 V, CE 28 V, CXP 10 V); m/z 269.2 [M+H]^+^→m/z 253.2 for formononetin (DP 190 V, FP 380 V, EP 14 V, CE 37 V, CXP 16 V); m/z 431.3 [M+H]^+^→m/z 269.2 for ononin (DP 80 V, FP 380 V, EP 13 V, CE 20 V, CXP 18 V); m/z 285.2 [M+H]^+^→m/z 270.2 for calycosin (DP 190 V, FP 380 V, EP 13 V, CE 33 V, CXP 17 V); m/z 447.2 [M+H]^+^→m/z 285.1 for Calycosin-7-O-β-D-glucoside (DP 80 V, FP 380 V, EP 12 V, CE 23 V, CXP 19 V); m/z 208.1 [M+NH_4_]^+^→m/z 173.2 for ligustilide (DP 70 V, FP 380 V, EP 14 V, CE 25 V, CXP 12 V); m/z 498.3 [M+NH_4_]^+^→m/z 179.2 for paeoniflorin (DP 76 V, FP 390 V, EP 14 V, CE 25 V, CXP 12 V) and m/z 798.6 [M+NH_4_]^+^→m/z 651.6 for digoxin (DP 20 V, FP 160 V, EP 10 V, CE 18 V, CXP 11 V, internal standard). Nine active constituents and the internal standard were separated by using a Phenomenex® Gemini C18 (150 mm×2.0 mm I.D, 5 µm particles) column maintained at ambient temperature. The following linear gradient program was applied to the analyte with the mobile phase consisting of methanol (0.1% formic acid, solvent A) and NH_4_OAc (0.1% formic acid, solvent B): 0–1 min: 30–70% A; 1–2 min 70–90%; 2–8 min: 90–90%; 8–9 min: 90–30%, 9–18 min: 30–30%, v/v. The sample injection volume was 10 µL. The flow rate was maintained at 0.2 µL/min.

### Preparation of calibration standards

The stock standard solution containing astragaloside I (1 mg/mL), astragaloside II (1 mg/mL), formononetin (1 mg/mL), ononin (1 mg/mL), calycosin (1 mg/mL), calycosin-7-O-β-d-glucoside (1 mg/mL) and paeoniflorin (1 mg/mL) was prepared in methanol. The standard powder of astragaloside IV was dissolved in 50% (v/v) acetonitrile to make a 1 mg/mL stock solution, and ligustilide was prepared in 100% acetonitrile for a 10 mg/mL stock solution. The internal standard stock solution of 1 mg/mL was also dissolved in 50% (v/v) acetonitrile. All solutions were stored at −20°C before use. These solutions were further diluted to give a serial of working standard solutions and samples for calibration in plasma were prepared by spiking aliquots of the stock solutions into drug-free plasma samples to obtain final concentrations in the range of 0.5–100 ng/mL for astragaloside I, astragaloside II, astragalosdie IV, ononin; 5–1000 ng/mL for formononetin; 5–100 ng/mL for calycosin; 1–100 ng/mL for calycosin-7-O-β-d-glucoside, ligustilide and 0.5–1000 ng/mL for paeoniflorin, and then processed according to the procedures described in Section 2.4.2. The validation samples were also prepared in the same way (1, 10, 100 ng/mL for astragaloside I, astragaloside II, astragalosdie IV, ononin calycosin-7-O-β-d-glucoside, ligustilide; 10, 100, 1000 ng/mL for formononetin, calycosin, paeoniflorin) at low, middle and high concentrations.

### Method validation

Full validation of the analytical method evaluated in this study was according to the US Food and Drug Administration guidelines for validation of bioanalytical methods [20]. Full validation was performed for determination of analyte samples in this study. Specificity, sensitivity, linearity, precision, accuracy, recovery, matrix effect, and stability were evaluated during the method validation.

The specificity was assessed by confirming the blank plasma extract for the presence or absence of interference as well as the lot-to-lot variation regarding interference. Sensitivity of analytes was determined by calculating the signal-to-noise ratio of lowest limit of quantitation (LLOQ) samples. The limits of detection (LOD) and quantification (LOQ) were determined at S/N (the ratio of signal to noise) of 3 and 10 under the present chromatographic conditions. The calibration standard curves were obtained by least-squares linear regression of the peak area versus the concentrations. All calibration curves were required to have a correlation value of at least 0.995. The concentration of each sample was derived from the calibration curve and corrected by the respective dilution volume. For a standard curve, the ratios of the chromatographic peaks area (analytes/internal standard) as ordinate variables were plotted versus the concentration of these drugs as abscissa. The samples in six replicates on the same day (intra-day) and on six successive days (inter-day) were prepared in the same manner to verify the precision and accuracy of the analytical method. The accuracy was calculated from the nominal concentration (Cnom) and the mean value of observed concentration (Cobs) as follows: accuracy (bias, %) = [(Cnom−Cobs)/Cnom]×100. The precision (relative standard deviation, RSD) was calculated from the standard deviation and observed concentration as follows: precision (RSD, %) = [standard deviation (SD)/Cobs]×100. The inter-day and intra-day accuracy and precision value for the lowest acceptable reproducibility concentrations were defined as being within ±15%.The LLOQ was defined as which the signal/noise ratio was ≧10 and the accuracy and precision were within 80–120%.

The matrix effect was determined at low, medium and high concentrations using three replicates as the ratio of the mean peak area of an analyte spiked post-extraction (set 2) to the mean peak area of the same analyte standards (set 1)×100%. The extraction recovery of analytes were determined at three concentrations by calculating as the ratio of the mean peak area of an analyte spiked before extraction (set 3) to the mean peak area of an analyte spiked post- extraction (set 2)×100%.

Stability was also assessed using the low, medium and high concentrations of analytes. Freeze-thaw stability was assessed over three freeze-thaw cycles. Short-term stability was determined by placing the samples at room temperature for 6 h. Long-term stability was evaluated by analyzing samples kept at −20°C for 14 days. Autosampler stability was determined by maintaining the samples at the autosampler temperature (8°C) for 12 h. All stabilities were calculating as the ratio of average concentration and freshly prepared samples.

### Pharmacokinetic application

Pharmacokinetic parameters including maximum plasma concentration (Cmax), time to reach the maximum concentrations (Tmax), half-life (t_1/2_), area under concentration–time curve (AUC) and oral clearance (CL) were estimated by a non-compartmental analysis using the pharmacokinetic program, WinNonlin Standard Edition Version 1.1 (Scientific Consulting, Apex, NC, USA). All results were expressed as average mean ± standard deviation (S.D.). ANOVA was used to evaluate differences, and a value of P<0.05 was taken as statistically significant.

## Results and Discussion

### Chromatographic and tandem mass spectrometric condition

Owing to the complexity of traditional Chinese medicine, many analogues may be co-eluted during analyses. In order to develop a sensitive and accurate LC-MS/MS method for the determination of BYHWT active compounds in rat plasma, a triple quadruple mass spectrometer equipped with electrospray ionization (ESI) source is currently one of the most useful tools available for simultaneous quantification of herbal compounds because of its high sensitivity and selectivity. Qualification analysis was performed using an MRM system consisting of two parts: selecting the precursor ion (MS 1) and selecting a specific fragment of precursor ion (MS 2). These two devices generate a very specific and sensitive response for the selected analyte. Thus the integrated peak for the target compound could be monitored in a sample after a simple one dimensional chromatographic separation. The determination of nine active compounds and internal standard were performed by positive mode ESI-MS/MS since higher ion intensities were observed using positive relative to negative mode. To characterize the fragment ions of the investigated compounds, an electrospray interface with good sensitivity, fragmentation and linearity was optimized.

The astragaloside profiles of astragaloside I, astragaloside II and astragaloside IV in the Radix Astragali could be observed. However, due to the complexity of the extract and the similar structures of astragalosides, it has been noted that these peaks require careful identification and separation [21]. Astragaloside I produced a positive ion of m/s 886.6, corresponding to the [M+NH_4_]^+^ precursor ions. The succeeding product ion of the precursor ion produced a fragmentation pattern lead to an ion at m/z 143.2. in addition, the mass transit ion patterns m/z 844.5→143.2 and m/z 802.6→143.2 were selected to monitor astragaloside II and astragaloside IV, respectively.

For isoflavonoids, the chemical structure of formononetin is similar to that of calycosin, differing in the substituent group located on a ring with a hydroxyl group [22]. The behavior of calycosin fragmentation could be extended to other isoflavones; and the protonated isoflavone aglycones reveal that the loss of a methyl radical was the predominant fragmentation for the aglycones, due to the formation of very stable cation radical structures. The major product ion of m/z 253.2 for formononetin may be due to the loss of a hydroxyl group. The compounds ononin and calycosin-7-*O*-β-d-glucoside are *O*-glycosides. In positive ion mode, the glycosidic bond of *O*-glycosidies were easily cleaved to produce daughter ions of [M+H−162]^+^ at 269.2 and 285.1 by the facile neutral loss of a glycoside residue from the corresponding protonated molecule ions at 431.3 and 447.2, respectiviely. The major product ions of ligustilide in Radix Angelicae Sinensis and Rhizoma Ligustici Chuanxiong at 173.2 corresponded to one hydroxyl group loss from the precursor ion. The major product ion of m/z 179.2 for paeoniflorin in Radix Paeoniae Rubra may be due to the loss of a glucose molecule, a benzoic acid molecule and a hydrone molecule [23]. The main fragments of the BYHWT active compoment are listed in Table 1. The MS/MS parameters manually obtained the highest response for each of the precursor and product ion combinations.

**Table 1:** The analytical condition of LC-MS/MS for the identification of the nine compounds and internal standard.

Compounds	Molecular weight	RT (min)	Mass fragments	
			Q1 Mass (amu)	Q3 Mass (amu)
Astragaloside I	869.04	11.21	886.6 [M+NH_4_]^+^	143.2
Astragaloside II	827.01	10.73	844.5 [M+NH_4_]^+^	143.2
Astragaloside IV	784.97	11.18	802.6 [M+NH_4_]^+^	143.2
Formononetin	268.26	8.32	269.2 [M+H]^+^	253.2
Ononin	430	8.23	431.3 [M+H]^+^	269.2
Calycosin	284.2	8.72	285.2 [M+H]^+^	270.2
Calycosin-7-O-β-D-glucoside	446.4	7.68	447.2 [M+H]^+^	285.1
Ligustilide	190.24	10.05	208.1 [M+NH_4_]^+^	173.2
Paeoniflorin	480.47	7.37	498.3 [M+NH_4_]^+^	179.2
Digoxin (IS)	780.94	9.12	798.6 [M+NH_4_]^+^	651.6

**Figure 2:**
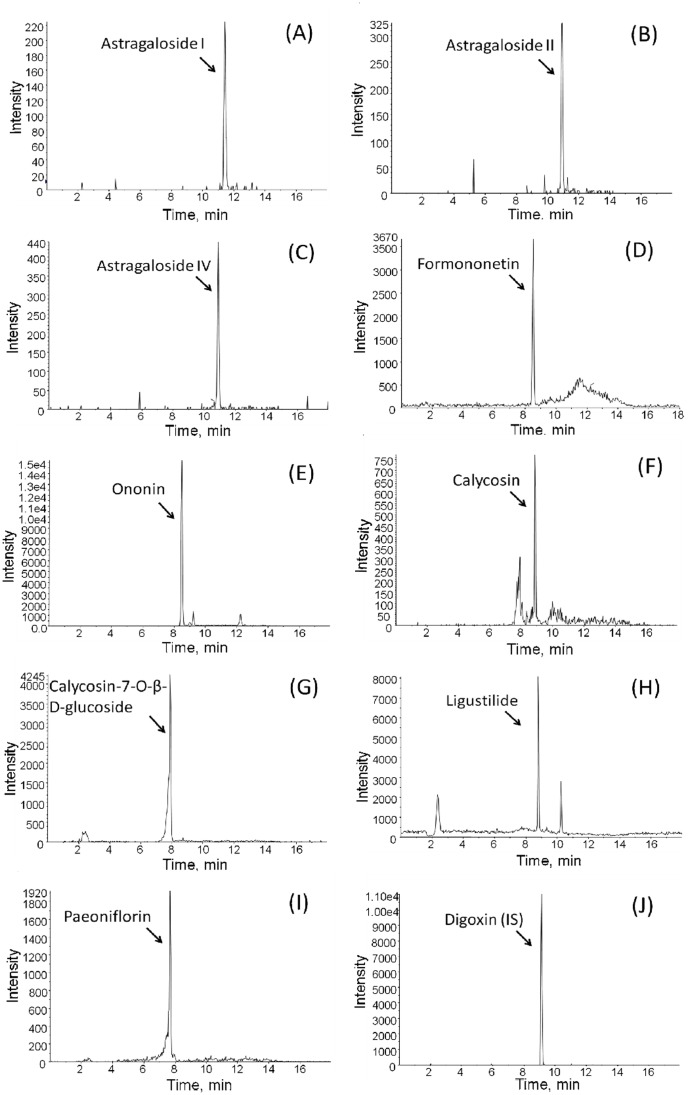
Typical MRM chromatograms of rat plasma sample after administration of bu-yang-huan-wu-tang extract: (A) Astragaloside I; (B) Astragaloside II; (C) Astragaloside IV; (D) Formononetin; (E) Ononin; (F) Calycosin; (G) Calycosin-7-O-β-D-glucoside; (H) Ligustilide; (I) Paeoniflorin and (J) Digoxin (IS).

In this developed method, the degree of interference was assessed by inspection of MRM mode. No significant interfering peaks from the plasma were found at the retention time and the ion channel of either the analytes or internal standard. Hence the development of the chromatographic system was concentrated on short retention times substituted for chromatographic separation. As shown in Fig. 2, the 9 selected marker compounds with IS cover a broad polarized range. The gradient elution, proper column and flow rate were pivotal influences on separation for multi-compounds. The chromatographic conditions achieved good resolution and appropriate ionization in the presence of endogenous species and co-elution. The chemical structures of astragalosides and internal standard have been noted as being relatively stable to acids [24]. To improve the sensitivity, adding acid to the mobile phase to enhance the ionization of astragalosides has also been suggested. It was also discovered that addition of 0.1% formic acid in the mobile phase provided a higher response and better peak sensitivity. In the course of the current study, several mobile phase systems, such as acetonitrile-water and methanol-water, in different ratios were examined. The acetonitrile-water system provides good separation for the investigated compounds, but the ligustilide peak had serious interference in the chromatogram. Finally, methanol- NH_4_OAc with 0.1% formic acid was employed, providing an appropriate retention time and low background noise, and was thus selected as the mobile phase system.

Ideally, a compound with similar physical-chemical properties should be used as the internal standard for analysis. Digoxin, which has a chemical structure similar to astragaloside I, astragaloside II and astragaloside IV, was chosen as the internal standard in the quantitative method in plasma. This compound was consistent with investigated analytes in chromatographic recovery and ionization properties. With extensive separation and optimization, the conditions established for reference compounds could be eluted with baseline separation in approximately 18 min. Under the proposed conditions, the electrospray ion mass spectrometer was used for quantitative analysis

### Linearity, limit of determination (LOD) and limit of quantification (LOQ)

The linearity of calibration curves were demonstrated by the good determination of coefficients (r^2^) obtained for the regression line. Good linearity was achieved over the calibration range, with all coefficients of correlation greater than 0.995. All samples were freshly prepared from the same stock solution, except for the standard solution. All the linear regressions of astragaloside I, astragaloside II, astragaloside IV, formononetin, ononin, calycosin, calycosin-7-O-β-d-glucoside, ligustilide and paeoniflorin in rat plasma displayed good linear relationships over the range mentioned in section 2.6. The mean values of regression equation of the analytes in rat plasma were: y = 0.0027x−0.0003 (r2 = 0.998, astragaloside I), y = 0.0039x+0.0002 (r2 = 0.999, astragaloside II), y = 0.0044x−0.0012 (r2 = 0.999, astragaloside IV), y = 0.0046x+0.0271 (r2 = 0.998, formononetin), y = 0.1458x−0.0001 (r2 = 0.999, ononin), y = 0.0059x+0.0196 (r2 = 0.998, calycosin), y = 0.0671x−0.0461 (r2 = 0.999, calycosin-7-O-β-D-glucoside), y = 0.0251x−0.0006 (r2 = 0.999, ligustilide) and y = 0.0278x+0.0054 (r2 = 0.9972, paeoniflorin).

The data showed that the LOD for the herbal ingredients in rat plasma were astragaloside I (0.1 ng/mL), astragaloside II (0.1 ng/mL), astragaloside IV (0.1 ng/mL), formononetin (1 ng/mL), ononin (0.1 ng/mL), calycosin (1 ng/mL), calycosin-7-O-β-D-glucoside (0.5 ng/mL), ligustilide (0.5 ng/mL) and paeoniflorin (0.1 ng/mL). Peak areas in chromatograms for the spiked plasma samples containing the above lowest concentrations were compared with the signal-to-noise ratio ≥3. Sensitivity was evaluated by the LOQ determinations, which are defined as the lowest concentration that can be reliably and reproducibly measured in at least three replicates. The data showed that the LOQ for the herbal ingredients in rat plasma were astragaloside I (0.5 ng/mL), astragaloside II (0.5 ng/mL), astragaloside IV (0.5 ng/mL), formononetin (5 ng/mL), ononin (0.5 ng/mL), calycosin (5 ng/mL), calycosin-7-O-β-D-glucoside (1 ng/mL), ligustilide (1 ng/mL) and paeoniflorin (0.5 ng/mL). Peak areas in chromatograms for the spiked plasma samples containing the above lowest concentrations was compared with the signal-to-noise ratio ≥10.

### Precision and accuracy

The precision and accuracy of the method were assessed in plasma by performing replicate analyses of spiked samples against calibration standards. The procedures were repeated on the same day and between six different days on the spiked samples with low, middle and high concentrations. The intra-day and inter-day precision and accuracy of the method are shown in Table 2. The precisions (relative standard deviation; RSD) were all less than 10%. The data indicated that the precision and accuracy of this method were acceptable.

**Table 2:** Intra-day and inter-day precision and accuracy for the determination for nine compounds from the assay samples (n = 6).

Nominal concentration (ng/mL)	Intra-day			Inter-day		
	Observed concentration (ng/mL)	Precision (%)	Accuracy (%)	Observed concentration (ng/mL)	Precision (%)	Accuracy (%)
Astragaloside I						
1	1.14±0.1	5.3	4.2	0.92±0.2	6.1	1.1
10	11.2±2.7	4.2	−1.9	11.1±2.9	4.3	−1.3
100	99.4±6.8	4.1	−3.3	99.8±7.1	4.6	0.8
Astragaloside II						
1	1.22±0.3	7.2	2.2	1.1±0.8	5.5	1.3
10	10.3±3.0	5.5	−1.8	11.0±3.1	3.2	−1.2
100	102±6.4	4.9	1.5	101±5.4	2.8	0.2
Astragaloside IV						
1	1.31±0.3	3.9	1.4	9.91±0.3	7.9	1.9
10	11.0±2.3	4.1	2.8	11.2±2.1	3.0	0.2
100	98.4±4.4	2.5	1.9	102±4.6	1.7	1.8
Formononetin						
10	11.0±2.3	5.6	2.7	9.37±1.9	2.8	3.2
100	99.1±4.2	4.9	1.3	98.6±5.5	4.3	−1.1
1000	998±18.2	2.9	1.8	1002±16.2	3.1	0.4
Ononin						
1	0.95±0.4	3.6	2.7	0.93±1.7	4.9	0.9
10	10.5±2.8	2.8	−1.1	10.5±2.1	3.4	−0.2
100	98.8±3.2	2.9	0.4	99.2±2.7	2.9	1.3
Calycosin						
10	10.2±1.1	6.4	2.5	9.67±1.3	5.4	4.8
100	101±5.2	4.1	1.0	99.3±4.6	2.1	2.1
1000	1005±7.1	4.8	0.9	1004±9.8	3.7	1.9
Calycosin-7-O-β-D-glucoside						
1	1.11±0.7	5.2	2.3	0.96±0.4	2.6	1.8
10	10.8±2.1	3.3	−1.1	10.2±1.6	3.3	−0.7
100	98.4±5.7	2.8	2.8	101.4±43.3	1.2	0.3
Ligustilide						
1	0.92±0.4	4.9	0.3	1.03±0.2	3.8	2.1
10	10.2±2.1	0.5	2.4	9.43±1.7	4.1	−0.9
100	97.3±5.5	3.6	−0.4	98.3±5.6	1.7	1.1
Paeoniflorin						
10	9.60±0.77	5.7	−1.1	11.3±0.4	0.9	1.1
100	97.3±7.64	7.4	0.2	10.8±3.9	−0.6	−0.3
1000	1008±42.5	4.7	0.3	1006±16	0.1	−3.6

### Matrix effect and recovery

The extraction recoveries and matrix effect from rat plasma are shown in Table 3. The one-step extraction procedure was fairly rapid. The protein precipitation by the organic solvent, acetonitrile (0.1% formic acid) was used to prepare the plasma samples for LC–MS/MS. Co-eluting compounds may enhance or suppress the ionization of the analytes. Owing to the sample preparation of the biological matrix (protein precipitation), matrix effects were expected. In order to avoid these matrix effects, good separation of the analytes was pursued. To measure the matrix effect, it was determined at three different concentrations for all analytes. An absolute matrix effect was observed for some of the analytes at some of the low, middle and high concentrations. Nevertheless, no relative matrix effects were seen, since the coefficients of variation (CV %) at each concentration level were <15% for all compounds. The extraction recoveries were also determined for three replicates from rat plasma spiked with low, middle and high concentrations of the nine analytes and internal standard. The mean recoveries of most samples were within 80–120%, except for astragaloside II. The recoveries of astragaloside II were 79.9±12% and 76.3±5.7% at concentration of 10 ng/mL and 100 ng/mL. Even it was slightly out of range, the extraction recovery of astragaloside II was stable and there was no other significant interference over the range.

**Table 3:** Extraction recovery and matrix effect of nine compounds and internal standard in rat plasma.

Compounds	Set 1^a^	Set 2^b^	Set 3^c^	Matrix effect (%)^d^	Recovery (%)^e^
Astragaloside I					
1	1.08±0.1	1.03±0.4	1.08±0.4	95.5±12	105±6.2
10	10.2±1.2	8.67±2.8	7.62±1.2	85.0±7.6	87.9±12
100	99.7±5.3	74.3±7.5	77.3±5.8	74.5±1.5	104±14
Astragaloside II					
1	1.24±0.2	1.00±0.1	0.88±0.3	80.9±7.4	87.4±6.0
10	10.8±0.6	9.63±1.5	7.70±2.0	89.2±6.8	79.9±12
100	101±3.2	80.9±6.3	61.7±4.9	80.1±13	76.3±5.7
Astragaloside IV					
1	1.28±0.2	1.16±0.5	1.12±0.3	90.4±1.4	96.5±2.6
10	9.81±1.3	11.1±2.4	10.8±3.2	113±4.3	97.7±9.4
100	98.2±5.8	105±4.2	99.4±3.9	107±8.1	94.6±9.4
Formononetin					
10	11.4±2.3	12.0±1.2	10.5±2.1	105±6.9	87.9±12
100	101±4.2	99.1±5.3	92.1±6.0	98.1±5.6	92.9±9.5
1000	995±11	918±14	898±20	92.3±5.9	97.8±0.2
Ononin					
1	0.98±0.2	0.93±0.3	0.86±0.1	94.8±7.5	92.2±6.0
10	11.2±1.3	10.6±2.9	10.6±2.2	94.9±9.5	100±2.6
100	97.9±5.2	96.4±6.3	90.8±3.9	98.5±8.2	94.2±8.7
Calycosin					
10	11.1±2.2	12.3±2.3	11.7±1.8	111±3.3	95.1±3.3
100	97.8±4.4	105±5.8	97.9±6.3	107±8.0	93.5±2.3
1000	997±8.8	1056±20	1056±23	106±10.1	100±0.2
Calycosin-7-O-β-D-glucoside					
1	1.19±0.3	0.95±0.4	0.83±0.2	80.0±8.7	87.6±8.3
10	9.86±1.3	9.69±2.1	9.39±1.4	98.3±8.2	96.9±0.9
100	98.7±4.3	97.9±6.7	93.8±8.2	99.2±4.4	95.8±7.1
Ligustilide					
1	0.97±0.5	0.93±0.1	0.90±0.3	95.7±10	97.3±3.1
10	11.4±1.1	11.8±2.7	11.3±0.4	103.8±5.7	95.5±9.9
100	99.3±7.1	98.8±9.3	95.1±6.2	99.5±7.5	96.2±1.9
Paeoniflorin					
10	10.2±1.7	10.4±2.3	11.0±2.9	102±9.0	106±5.2
100	98.8±3.9	98.8±6.2	94.7±4.6	100±7.7	95.8±4.2
1000	995±17	991±11	966±15	99.6±1.4	97.5±3.8
Digoxin (IS)					
66.7	64.3±4.4	65.5±5.3	65.6±4.7	102±5.2	100±4.2

Data are expressed as means ± S.D. (n = 3).

a Neat is the standard in injection solvent.

b Post-spiked is the standard spiked in the prepared control plasma.

c Pre-spiked is the standard spiked into control plasma before prepared.

d Matrix effect (%) calculated as [(b/a)×100]%.

e Recovery (%) calculated as [(c/b)×100]%.

### Stability

To estimate the stability of extracted samples during the experiments, storage, and sample preparation processes were designed and conducted under different conditions, including freeze-thaw cycle analysis, short-term stability, long-term stability and autosampler stability by analysis using biological samples. Deviation of the mean measured concentrations of the samples from the nominal concentration was within 15%. All established stabilities for nine active compounds are shown in Table 4. The stability of freeze-thaw cycle analysis, short-term and autosampler remained generally stable under the analyte storage and analytical process conditions. The long-term tests showed little variation compared to the initial concentration in astragaloside I (100 ng/mL), calycosin (100, 1000 ng/mL), Calycosin-7-O-β-D-glucoside (10, 100 ng/mL), ligustilide (100 ng/mL) and paoniflorin (10, 1000 ng/mL). These observations indicate that testing must be executed after the sample extraction. Based on the above method validation, the processes for the analysis of these herbal ingredients were acceptable.

**Table 4:** Stability of the nine compounds in rat plasma samples.

Concentration (µg/mL)	Stability (%)			
	Freeze-thaw stability	Short-term stability	Long-term stability	Autosampler stability
Astragaloside I				
1	4.6±3.6	−3.6±8.0	−14±17	−5.3±7.9
10	3.8±2.7	−4.1±10	−8.3±2.3	4.3±6.6
100	−1.8±5.4	−6.4±6.4	−27±12	−4.9±7.0
Astragaloside II				
1	4.4±3.6	2.4±12	8.7±10	13±10
10	−2.3±0.8	−1.3±1.9	−0.8±16	−0.3±11
100	0.4±1.2	−6.8±5.7	1.7±21	−5.4±13
Astragaloside IV				
1	−3.2±5.0	12±15	−17±5.3	3.8±9.8
10	−4.1±3.4	4.7±6.2	−6.3±8.8	3.3
100	6.1±0.4	1.6±9.1	2.1±13.5	−8.6±12
Formononetin				
10	−4.9±3.8	−3.4±12	−13±5.5	−13±4.1
100	5.8±10	1.1±10	−12±7.5	−4.6±11
1000	−3.5±6.0	1.5±10.4	1.0±6.4	0.8±12
Ononin				
1	3.9±6.4	12±6.3	−7.2±10	4.7±7.7
10	−2.5±7.3	−12±4.6	−6.2±2.9	−7.0±1.4
100	4.2±1.3	6.7±2.1	3.2±3.9	8.1±3.8
Calycosin				
10	9.6±4.7	1.4±11	2.5±5.7	−3.5±11
100	8.6±3.8	14±12	17±5.2	−2.2±3.5
1000	2.9±4.5	13±7.3	21±12	7.1±4.9
Calycosin-7-O-β-D-glucoside				
1	5.8±6.1	2.5±11	6.4±12	−8.4±12
10	4.9±3.5	7.3±12	23±13	2.1±9.1
100	7.0±1.2	−0.1±0.9	20±12	3.5±7.9
Ligustilide				
1	8.7±5.9	14±11	6.7±10	2.0±10
10	2.7±2.2	11±10	5.0±14	13±7.2
100	6.5±1.4	12±7.5	23±12	−1.6±10
Paeoniflorin				
10	7.3±4.6	7.7±10	21±15	−3.3±7.3
100	2.1±1.8	−1.8±9.4	10±7.3	−1.6±8.2
1000	9.5±0.2	11±8.4	27±9.9	8.5±5.1

Data are expressed as means ± S.D. (n = 3).

### Application of the analytical system in pharmacokinetics study

**Figure 3:**
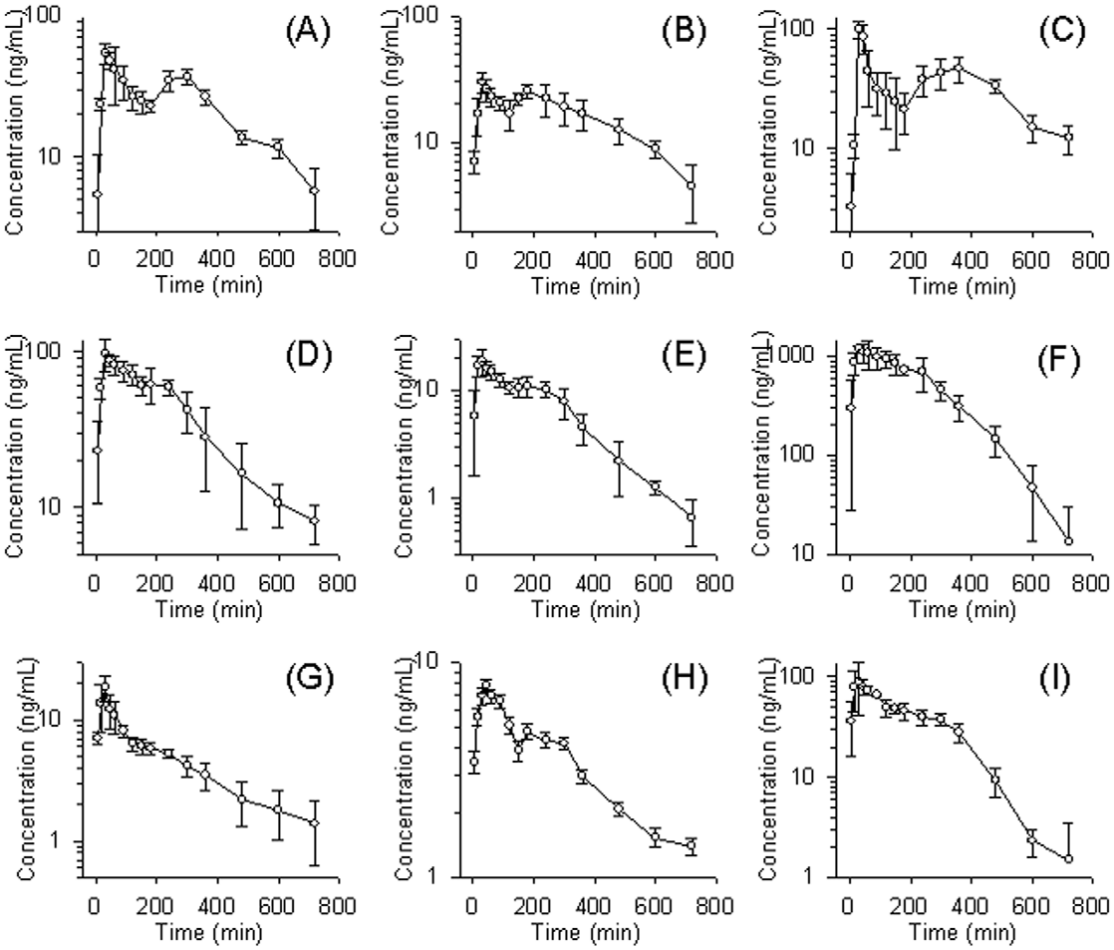
Mean plasma concentration–time profile in rat plasma after oral administration of bu-yang-huan-wu-tang extract for astragaloside I (A), astragaloside II (B), astragaloside IV (C), formononetin (D), ononin (E), calycosin (F), calycosin-7-O-β-D-glucoside (G), ligustilide (H) and paeoniflorin (I).

The validated HPLC-MS/MS method was successfully applied for the simultaneous determination of nine active constituents for 12 h after BYHWT extract administration (10 g/kg, p.o.) in the rat. The mean plasma concentration-time profiles are illustrated in Fig. 3 and the pharmacokinetic parameters are presented in Table 5.

**Table 5:** Pharmacokinetic parameters of the nine compounds in rat after oral administration of bu-yang-huan-wu-tang.

Parameter	C_max_ (ng/mL)	T_max_ (min)	t_1/2_ (min)	AUC (min mg/mL)	Cl (mL/min/kg)
Astragaloside I	55±7.8	40±16	166±28	16.6±2.6	90.5±14
Astragaloside II	30±4.9	37±6.8	212±14	11.4±2.3	129±15
Astragaloside IV	100±16	35±7.7	170±8.3	23.0±5.7	64.3±13
Formononetin	98±17	38±8.2	175±13	26.7±6.0	57.4±14
Ononin	19±4.5	35±7.7	134±9.6	4.6±1.0	351±47
Calycosin	1231±171^a^	40±16	75±11	308±73^b^	5.4±1.3
Calycosin-7-O-β-D-glucoside	18±4.1	32±6.1	338±132	3.25±0.7	428±143
Ligustilide	7.8±0.2	45±0.0	267±1.2	2.43±0.06	532±11
Paeoniflorin	97±33	35±7.7	93±32	21.0±3.9	76.5±15

Data expressed as mean ± standard error. (n = 6) Cmax: the maximum plasma concentration, t_1/2_: half-life, AUC: area under the concentration-time curve, Cl: clearance.

a Significantly different (p<0.05) from the other active compounds in C_max_.

b Significantly different (p<0.05) from the other active compounds in AUC.

The concentration-time curves of astragaloside I, astragaloside II, astragaloside IV and ligustilide presented the phenomenon of a double-peak absorption phase in the plasma profile, and the other analytes seemed to have the same tendency. However, the other herbal ingredients of formononetin, ononin, calycosin, calycosin-7-O-β-d-glucoside and paeoniflorin presented a single and plateau absorption phase, consistent with a previous report [15]. By means of plateau absorption or multiple-peak absorption phase, these phenomenona were similar to another report on Chinese herbal compounds [25]. There were many possible explanations that attempt to explain the phenomenon of multiple-peak behavior. Similar multiple peak phenomena have been observed for a number of other oral drugs [26] [27]. Several mechanisms have been proposed for the phenomenon: 1) enterohepatic recycling, the cycle in which bile salts and drugs excreted by the liver are absorbed by the intestinal mucosa and returned to the liver via the portal circulation [28]. 2) the presence of absorption sites along the stomach and different gastrointestinal segments [29]. 3) variable gastric emptying; a previous report suggests that the drug may cause increased gastric pH which in turn increases the gastric emptying rate [30]. After increasing the gastric emptying rate, a substantial amount of drug emptied into the duodenum soon after the administration can cause a rapid increase in plasma concentrations, resulting in the disappearance of double peaks [31].

Herbal preparation is a complex formulation which contains several herbs and ingredients. It is possible that the different characteristics of physical-chemical properties for individual ingredients may affect each other. In an in vitro herbal analysis, it is indicated that the berberine content in herbal preparation of Huang-Lian-Jiee-Dwu-Tang (8.15±0.39 mg/g) was less in the single herb Coptidis Radix (53.63+2.45 mg/g) but higher in Phellodendri Cortex (3.02+0.22 mg/g) when extracted with water, which suggest that herbal matrix effects were involved in the multiple compositions of the herbal preparation [32]. Another in vivo experiment discussed the pharmacokinetic of single herbal ingredient, single herbal extract and herbal preparation. Rutaecarpine (25 mg/kg/day, p.o.) (the herbal ingredient of Evodia rutaecarpa), the ethanol extract of Evodia rutaecarpa (1 g/kg/day, p.o.) and multiple herbal preparation of Evodia rutaecarpa (Wu-Chu-Yu-Tang; 1 g/kg/day, p.o.) were individually pretreated daily for three consecutive days in rats and caffeine was administered (2 mg/kg, i.v.) on the fourth day. The results indicate that the rutaecarpine (single ingredient), extract of Evodia rutaecarpa, and herbal preparation (Wu-Chu-Yu-Tang) provide significant interaction for the pharmacokinetics of caffeine [33].

Rat is anatomically lack of gall bladder and the bile juice is directly excreted into duodenum. A previous biliary excretion report indicates that the gastrodin (an active substance isolated from *Gastrodia elata* Blume) and its metabolite have been detected in the bile duct [34]. Another pair-rats experimental model is designed to investigate the enterohepatic circulation of colchicine [35]. The results show that the AUC of colchicine was 847.7±141.0 and 55.5±29.2 min µg/mL in the donor and recipient rats, respectively. The biliary excretion and enterohepatic circulation may have significant effects on the pharmacokinetics of a number of drugs. Our data demonstrate that the double-peak phenomenon in the pharmacokinetic curve may be caused by the biliary excretion and enterohepatic circulation. However, this is the first reported case of double peaks for BYHWT absorption. The double-peak phenomenon caused by the hypothesized mechanism may have important therapeutic and drug interaction implications, especially because traditional medicines are commonly coadministered with other drugs. Furthermore, studies are needed to investigate the mechanism underlying the double-peak phenomenon for BYHWT with its active components in the future.

The cycloartane-type triterpene glycosides group in Radix Astragali of astragaloside I, astragaloside II and astragaloside IV, which were comprised of the same aglycone cycloastragenol and only differ in number and position of glucosyls, but their pharmacokinetic behaviors were significant different. The contents of astragaloside I, astragaloside II and astragaloside IV in the herbal preparation of BYHWT were 2.46, 0.69 and 0.54 mg/g, respectively. However, the values of C_max_ and AUC of astragaloside I and astragaloside II were much lower than astragaloside IV. This phenomenon might be referred to the biotransformation of astragaloside I to astragaloside IV about intestinal bacteria and enzymatic reaction, arising from great increase of astragaloside IV in vivo. Moreover, the constituents in herbal preparations may be the substrates of inhibitors, or inducers of Cytochromal enzymes, and hence had individual or combination effects on the pharmacokinetics of each other [36].

Calycosin displayed rapid and sustained absorption, with the largest C_max_ at 1231±171 ng/mL (p<0.05) and AUC at 308±73 min mg/mL (p<0.05); and at 5±11, calycosin had the shortest elimination half-life min among nine tested compounds of BYHWT. Formononetin had second largest amount of AUC value, perhaps due to its structural similarity to calycosin. Ononin and calycosin-7-O-β-d-glucoside had identical pharmacokinetic parameters in organisms, with their C_max_ and T_max_ being similar and both of them having low AUC. However, the BYHWT contents of ononin and calycosin-7-O-β-d-glucoside in were 0.63 and 2.56 mg/g, respectively, since the ononin content in plasma was higher. This might be ascribed to the relatively weak polarity of ononin.

Ligustilide is one of the most important bioactive components of Radix Angelicae Sinensis and Rhizoma Ligustici Chuanxiong, and paeoniflorin is the major bioactive ingredient in Radix Paeoniae Rubra in BYHWT. Significantly, the amounts of crude drug of Radix Angelicae Sinensis and Rhizoma Ligustici Chuanxiong in BYHWT were much higher than that of Radix Paeoniae Rubra. But there was less ligustilide than paeoniflorin found in the quantitative results, and our findings indicated that the C_max_ and AUC of ligustilide were much lower than paeoniflorin in a living organism. Moreover, the extent and rate of drug uptakes into the systemic circulation of AUC and C_max_ for ligustilide were the lowest in BYHWT. However, the previous report did not monitor ligustilide as one of their target components [15]. In this study, we provide more detailed information to enable better understanding for the pharmacokinetic profiles of the herbal preparation BYHWT. Moreover, based on the interpretation of pharmacokinetic profiles for the individual herbal ingredients, the pharmacokinetic characteristics of this complicated traditional Chinese herbal preparation might be gradually revealed.

## Conclusion

In present study, reliable high-performance liquid chromatography coupled with tandem mass spectrometry was successfully used for the quantitation of bioactive compounds of astragaloside I, astragaloside II, astragaloside IV, formononetin, ononin, calycosin, calycosin-7-O-β-d-glucoside, ligustilide and paeoniflorin in rat plasma after oral administration of BYHWT. These pharmacokinetic studies were performed through an experimental model of freely moving rats. The assay provided adequate recovery and matrix effect with good precision and accuracy. These pharmacokinetic results provide a constructive contribution to better understand the extensive action mechanism of absorption, distribution, metabolism and excretion, and to prepare for future clinical evaluation.
